# Increased levels of thymidine kinase 1 in malignant cell-derived extracellular vesicles

**DOI:** 10.1016/j.bbrep.2024.101761

**Published:** 2024-06-21

**Authors:** Ehsan Manouchehri Doulabi, Louise Dubois, Liza Löf, Tanay Kumar Sinha, George Mickhael Harinck, Per Stålhandske, Anders Larsson, Masood Kamali-Moghaddam

**Affiliations:** a Department of Immunology, Genetics & Pathology, Science for Life Laboratory, Uppsala University, SE-751 08, Uppsala, Sweden; b Department of Medical Sciences, Clinical Chemistry, Uppsala University, SE-751 85, Uppsala, Sweden; c Biovica International AB, Dag Hammarskjölds väg 54B, Uppsala Science Park, SE-752 37, Uppsala, Sweden

**Keywords:** Prostate cancer, Extracellular vesicles, p53, Prostasomes, Thymidine kinase 1 (TK1), Cancer, Diagnostics

## Abstract

Extracellular vesicles (EVs), whose main subtypes are exosomes, microparticles, and apoptotic bodies, are secreted by all cells and harbor biomolecules such as DNA, RNA, and proteins. They function as intercellular messengers and, depending on their cargo, may have multiple roles in cancer development. Thymidine kinase 1 (TK1) is a cell cycle-dependent enzyme used as a biomarker for cell proliferation. TK1 is usually elevated in cancer patients' serum, making the enzyme a valuable tumor proliferation biomarker that strongly correlates with cancer stage and metastatic capabilities. Here, we investigated the presence of TK1 in EVs derived from three prostate cancer cell lines with various p53 mutation statuses (LNCaP, PC3, and DU145), EVs from the normal prostate epithelial cell line RWPE-1 and EVs isolated from human seminal fluid (prostasomes). We measured the TK1 activity by a real-time assay for these EVs. We demonstrated that the TK1 enzyme activity is higher in EVs derived from the malignant cell lines, with the highest activity from cells deriving from the most aggressive cancer, compared to the prostasomes and RWPE-1 EVs. The measurement of TK1 activity in EVs may be essential in future prostate cancer studies.

## Highlights

• Thymidine kinase 1 (TK1) serves as a significant biomarker for cell proliferation and cancer progression, with elevated levels associated with higher metastasis level.
• TK1 enzyme activity is higher in small extracellular vesicles (sEV) derived from prostate cancer cell lines compared to the normal prostate epithelial cell line and prostasome.
• Measuring TK1 enzyme activity in sEV holds a potential as a diagnostic routine for evaluating prostate cancer aggressiveness.

## 1 Introduction

Extracellular vesicles (EVs) are lipid bilayer vesicles secreted by all cells, with mainly three subtypes, exosomes, microparticles (MVs), and apoptotic bodies. Exosomes with sizes of 30–150 nm in diameter are produced by invagination of the cell membrane to form endosomes in the cytoplasmic compartment of the cells, where they develop into multivesicular bodies (MVBs) that fuse with the plasma membrane of the cell to be actively released via exocytosis [1]. The MVs are 100 nm to 1 μm in diameter and formed via outward budding of the cell's plasma membrane [2]. Apoptotic bodies, 50 nm to 5 μm in size, are generated by dying cells, where the cell membrane is separated from the cytoskeleton [3]. Exosomes and MVs are involved in cell-to-cell communications [[4], [5], [6]], and as carriers of different molecules, they thus play a crucial role in several biological processes [7].

Differentiation between subtypes of EVs is still, to some extent, unclear due to their overlapping size and cargo [8]; however, in this study, we focus on small EVs (sEVs) of up to 200 nm in size.

In cancer, several critical processes, such as tumor invasion, metastasis of cancer cells, and tumor growth, are regulated via cell-to-cell communication. EVs and other nanoparticles are involved in this communication by exporting different molecules, such as proteins, peptides, and fragments of DNA and RNA, and convey functional information within the tumor microenvironment and mediate cell-to-cell communication to distant sites [9,10]. Depending on their cargo, EVs from malignant cells can promote tumor growth and metastasis by protecting the tumor cells from the immune system [11]. EVs may also promote angiogenesis, modify the microenvironment around the tumor, and influence therapy effects [12]. The prostate-specific EV, known as prostasomes, were the first described member of the exosome family [13], and they are shown to have, among many different functions, a vital role in the protection of sperm cells against the immunological defenses of the female reproductive tract [14].

Cell proliferation is one of the most important features of tumor aggressiveness and a common variable used to evaluate tumor progression and prognosis [15]. Thymidine kinase 1 (TK1 EC 2.7.1.21) is an essential enzyme in DNA synthesis and is a nucleotide salvage pathway enzyme involved in the homeostasis of the deoxythymidine triphosphate (TTP) pool used for DNA repair and rapid access during cellular DNA synthesis. The TK1 gene expression is upregulated during the S phase, preparing the cell for cell division. In highly proliferating cells, such as tumor cells, the TK1 enzyme activity is elevated, and its activity is considered an essential cellular proliferation tumor biomarker. The activity of the TK1 enzyme can be detected in both liquid and solid biopsies [16,17].

TK converts thymidine (Thd), salvaged from extracellular catabolic activity, to its monophosphate, deoxythymidine monophosphate (TMP), by transferring a γ-phosphate group from adenosine triphosphate (ATP) to the 5′ position of the nucleoside ribose unit. TMP is converted in two steps to TTP, which are then ready to be incorporated into DNA [18]. There are two isoenzymes of thymidine kinase: 1 and 2, of which TK1 is present in the cytosol. TK1 has high specificity for phosphorylating Thd, whereas the mitochondrial isoenzyme, TK2, supplies the organelle with TMP and deoxycytidine monophosphate (dCMP) nucleotides for mitochondrial DNA synthesis [19].

In the blood, elevated TK1 levels are associated with tumor progression. It may also be an indication of early events in carcinogenesis [20,21]. Elevated levels of TK1 in serum have been reported in early malignancy in several hematological cancers and solid tumors, including breast, lung, and melanoma cancers. High levels of TK1 enzyme activity in serum are also associated with cancer grade and stage. Therefore, TK1 activity in serum may serve as an early detection biomarker for several malignancies [[22], [23], [24], [25]]. A screening study of serum TK1 activity, comprising 11,880 individuals, revealed that 83 % of those having a TK1 activity level above the cut-off for healthy individuals had different diseases, from hepatitis to malignant cancers [26].

Weagel et al. found that active TK1 is localized to the membrane of malignant cells from malignancies such as lung, breast, and colon cancers. However, TK1 is localized solely in the cytosol [27] in regular, resting, or proliferating cells. These data suggest that TK1 could act as a cell surface biomarker for these malignancies [16]. In addition, it has been shown that TK expression is affected by cancer events such as loss of p53 tumor suppressor gene regulating capacity [28].

This study aimed to investigate TK1 enzyme activity in prostasomes isolated from seminal fluids and in sEVs of up to 200 nm derived from prostate cancer cell lines. To investigate any potential synergy between p53 and TK1, we investigated EVs derived from cell lines with regard to different metastatic activity [29,30].

Here, we investigate the TK1 enzyme activity in prostasomes from healthy individuals and sEVs from three malignant cell lines derived from prostate cancer. We demonstrate that the TK1 enzyme activity is higher in EVs derived from the malignant cell lines, with the highest activity from cells deriving from the most aggressive cancer, compared to the prostasomes from healthy individuals and prostate epithelial normal cell line.

## 2 Results

### 2.1 TK1 activity correlates to the cellular metastatic activity of EVs

The cell lines PC3, DU145, and LNCaP have previously been shown to have different levels of metastatic activity. While PC3 is highly metastatic, DU145 and LNCaP are shown to be moderate and poor in their metastatic activity, respectively [31,32], and the RWPE-1 is a regular prostate epithelial cell line [33] (Table 1).

**Table 1:** Cell lines with their p53 status and metastatic activity used in this study for production of sEVs.

Cell line	p53 status	Metastatic activity	Origin
**LNCaP**	Wild-type	Low	Prostatic adenocarcinoma
**PC3**	Null	High	Small cell carcinoma
**DU145**	Mutated	Moderate	Primary prostatic adenocarcinoma
**RWPE-1**	Wild-type	None (not malignant)	Epithelial prostate

EVs from these malignant and normal cell lines and prostasomes in seminal fluids from healthy males were purified according to optimized protocols described in the experimental procedure section. The quality of the purified cell line-derived EVs and prostasomes was examined by negative stain electron microscopy (EM), nanoparticle tracking analysis (NTA), and Western blot. Negative stain EM revealed structurally intact cell line-derived EVs and prostasomes (Supplementary Fig. 1). The size of the cell line-derived EVs and prostasomes was determined using NTA, where the main peak for both prostasomes and the cell-derived EVs was 100–200 nm (Supplementary Fig. 2). Western blot analysis revealed the presence of sEV markers TSG-101 and CD63 in EVs and cells and the absence of the ER marker calnexin, indicating the purity of the cell line-derived EV samples and prostasomes from protein contaminations. The presence of TK1 was also demonstrated in both cells- and EV lysates (Supplementary Fig. 3).

**Fig. 1:**
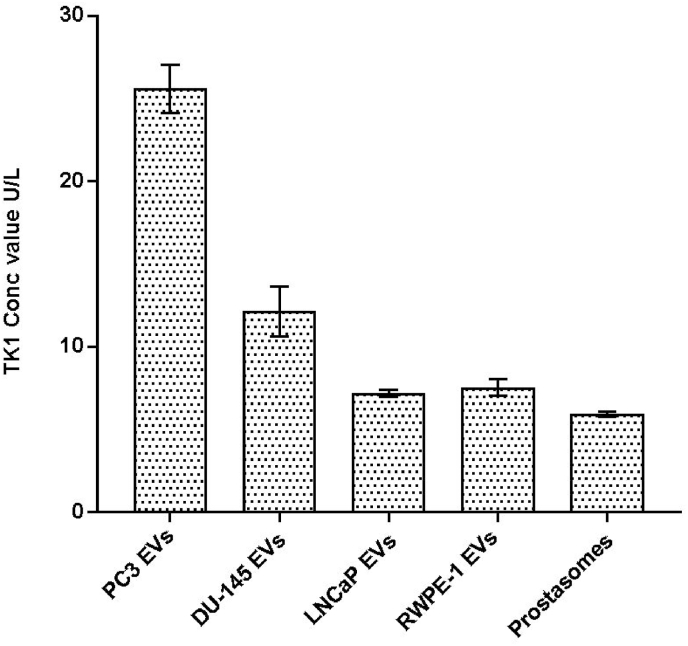
Measured TK1 activity for EVs isolated from the three cancer cell lines, PC3 DU145, LNCaP, from the normal epithelial prostate cell line RWPE-1, and prostasomes isolated from seminal fluids from healthy individuals. TK1 activities were normalized by the total protein concentration of 40 μg/mL for each sample. The mean values of TK1 activity for EVs isolated from the cell lines PC3, DU145, LNCaP, RWPE-1, and from prostasomes, 25, 12.1, 7.2, 7.5, and 5.9 U/L, respectively (*n* = 3, mean ± s.d.).

The TK1 activities were measured in the cell line-derived EVs and the prostasomes isolated from seminal fluids from healthy men. The activities were normalized based on the total protein concentration measured by Dot-It-Spot-It, with a total protein concentration of 40 μg/mL for each sample. The highest TK1 activity with a mean value of 25 U/L was observed for PC3-derived EVs. The mean values of TK1 activity for EVs isolated from the other three cell lines, DU145, LNCaP, RWPE-1, and from the prostasomes were measured to 12.1, 7.2, 7.5, 5.9 U/L, respectively (Fig. 1).

### 2.2 TK1 activity of purified PC3-derived EVs spiked in female plasma

**Fig. 2:**
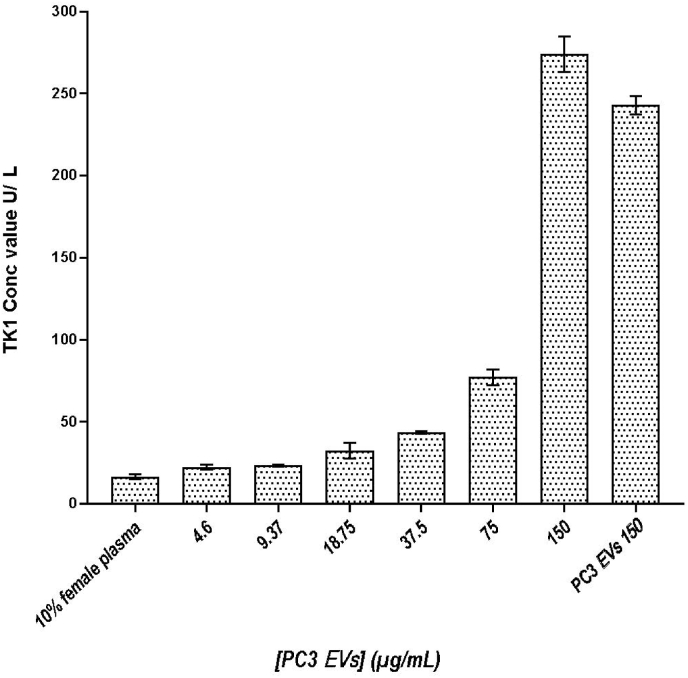
TK1 activity measurement from purified PC3-derived EVs at different protein concentrations, a 2-fold serial dilution with starting concentration at 4.6 μg/mL, spiked in 10 % female plasma (*n* = 3, mean ± s.d.). 10 % female plasma without spiked EVs and pure PC3-derived EVs at the highest protein concentration (150 μg/mL) were also analyzed.

**Fig. 3:**
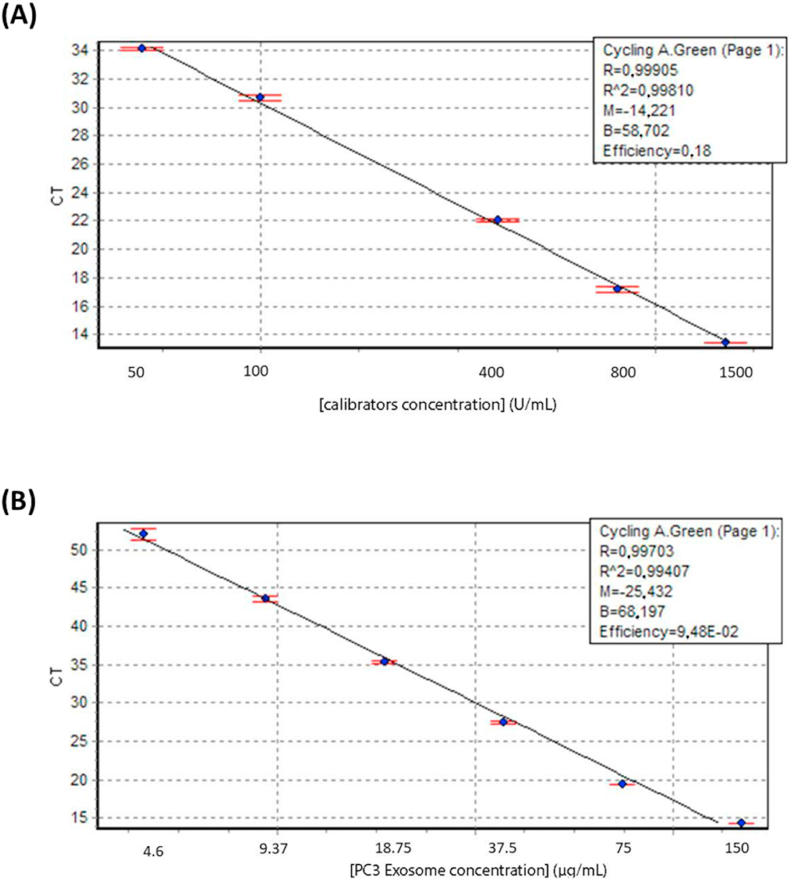
Top graph (**A**) displays the real-time assay control run of the calibrators A-D. The R^2^ > 0.998 indicates a usual assay run. In bottom graph (**B**), the real-time assay control was run with a 2-fold serial dilution of PC3 cell line EVs with an R^2^ > 0.994. Data are represented as mean ± s.d. (*n* = 3).

The EVs isolated from PC3 cell lines with the highest TK1 activity (Fig. 2) were selected as a positive control to evaluate our approach for the kinase activity measurements. A 2-fold serial dilution from 150 to 4.6 μg/mL PC3-derived EVs was spiked in 10 % female plasma in PBS. A sample with only 10 % female plasma in PBS was used as a negative control, while a sample with purified PC3-derived EVs was used as a positive control. The endogenous TK1 activity in the negative control with 10 % female plasma was measured at 16.7 U/L, while the TK1 activity recorded for most diluted EV samples of 4.6 μg/mL (approximately 5 ng total protein of EVs) was 22 U/L. Calibrators indicated a usual assay run with R^2^ > 0.998, and a 2-fold serial dilution of EVs, based on total protein concentration, was also run to investigate the quality of the dilutions with an R^2^ > 0.994 (Fig. 3).

### 2.3 Performance of TK1 real-time activity assay under different conditions

**Fig. 4:**
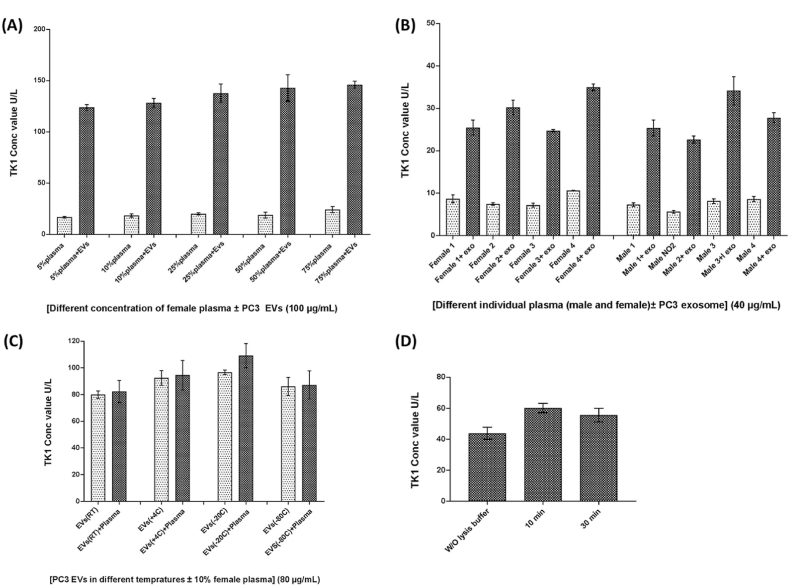
**(A),** TK1 activity measured in 100 μg/mL PC3-derived EVs spiked in pooled female plasma ranging from 5 % to 75 % in PBS. **(B)** The background levels of the TK1 activity were analyzed in 10 % plasma samples from four men and four women with and without 40 μg/mL spiked PC3-derived EVs. **(C)** Analysis of the TK1 activity in 80 μg/mL of the PC3-derived EVs in PBS or 10 % plasma after storing at RT, 4 °C, −20 °C and −80 °C for 24 h. **(D)** The TK1 activity was investigated in 80 μg/mL PC3-derived EVs that were treated with or without lysis and at either 10 or 30 min of lysis incubation. Data are represented as mean ± s.d. (*n* = 3).

To evaluate the method's robustness, the TK1 activity of the sEVs was measured under different conditions, such as various plasma concentrations, storage temperature, and the effect of different lysis conditions. First, the assay recovery was examined by measuring the TK1 activity of 100 μg/mL PC3-derived sEVs spiked in pooled plasma from females ranging from 5 % to 75 % in PBS. No significant difference in TK1 activity could be detected in the samples with different plasma concentrations. This indicates that a more concentrated matrix, such as up to 75 % plasma, does not interfere with the analysis (Fig. 4A).

Next, the background levels of the TK1 activity were analyzed in 10 % plasma samples from four males and four females with and without spiked 40 μg/mL PC3-derived EVs. We could detect TK1 activity with individual differences in the samples before the spiking of EVs. The levels of the TKI activity increased in all samples after the spiking of PC3-derived EVs, indicating that individual TK1 activity does not interfere with the analysis (Fig. 4B). To investigate whether the measurement of TK1 activity in sEVs is affected by sample treatment under different conditions, various storing temperatures of sEVs were tested. Eighty μg/mL of the PC3-derived EVs in either PBS or in 10 % plasma were stored at RT, 4 °C, −20 °C and −80 °C for 24 h. No significant differences in the TK1 activity levels could be observed for the tested different storage conditions (Fig. 4C). Finally, the TK1 activity was investigated in sEVs that were treated with or without lysing buffer and at different time conditions for the lysing treatment. There was no significant difference between TK1 activity in the samples treated for 10 min and those treated for 30 min. Moreover, a comparable level of high TK1 activity was observed in samples not subjected to lysis (Fig. 4D). This observation indicates the potential presence of TK1 on the surface of sEVs.

## 3 Discussion

TK1 has a crucial role in maintaining a steady state of TTP inside the cell to support the cell in synthesizing DNA and S phase duplication. The TK1 enzyme is a valuable tumor biomarker as it reflects the rate of cellular proliferation [34]. It can thus be used as a general indicator of tumor growth, particularly for assessing tumor aggressiveness. Serum TK1 enzyme activity is a clinically valuable biomarker, and the management of hematological malignancies has been particularly well-studied [35]. Upregulated TK1 enzyme activity in serum has been reported in many patients with solid tumors, including thyroid carcinoma [36]; gastric [37]; ovary [38]; prostate and breast cancers [39]. The detection of serum TK1 enzyme activity is sensitive and specific enough to predict early-stage and advanced lung cancer [40].

One of the mediators induced in response to DNA damage and cell cycle arrest is the protein p53, the product of the tumor suppressor gene *tp53*. Approximately 50 % of human cancers harbor alterations or mutations in *tp53* [41,42]. In cancer events and upon loss of p53 regulating capacity, it has been shown that p53 affects the regulation of TK1 expression. TK1 is drastically upregulated in p53 deficient cells [29,30]. In a study by Wang and Wahl [43], using a whole-genome microarray profile, they compared the expression levels of the critical pathways involved in proliferation in different prostate cell lines. They demonstrated that the gene expression of TK1, from high to low expression, were PC3 (1104 %), DU145 (934 %), and LNCaP (727 %) as compared to an average of gene expression in regular prostate cell lines [43]. Our findings support the notion of increased TK1 activity in sEVs derived from PC3 and DU145 cell lines compared to that in sEVs from the normal epithelial prostate cell line RWPE-1 and SF-EVs from healthy individuals. The Western blot analysis revealed a higher concentration of TK1 protein in PC3 cell lysates compared to LnCap and DU145 cell lysates, corroborating the notion of PC3's more malignant phenotype relative to the other cell lines. The elevated TK1 concentration in PC3 cell lysates suggests a potential association between TK1 levels and the aggressiveness of PC3 cells, thereby implicating TK1 as a potential marker for malignancy. These observations contribute to our understanding of the role of TK1 in prostate cancer and its potential as a diagnostic or prognostic indicator. We also observed differential patterns of TK1 between sEVs and cell lysates on the Western blot, which can be attributed to multiple factors (Supplementary Fig. 3).

TK1 may be actively sorted and concentrated into sEVs, resulting in higher levels of TK1 in the sEV fraction compared to the whole-cell lysate. Additionally, differences in subcellular localization and enrichment of TK1 in sEVs versus the total cellular content can contribute to the observed pattern. Post-translational modifications occurring in sEVs may also impact TK1's mobility and migration pattern on the blot. Elevated serum thymidine kinase 1 (TK1) is a significant risk factor indicating a high proliferation potential of tumors at the time of excision. It is recognized as a valuable cancer marker in various solid tumors [20,44]. Our Western blot data shows a higher concentration of TK1 in PC3 cell lysates compared to LnCap and DU145, supporting the notion of PC3's heightened malignancy. While the correlation between TK1 activity and protein concentration in sEVs was not explored in this study, the established link between TK1 activity and tumor proliferation potential suggests that investigating this association may offer insights into TK1's diagnostic potential in prostate cancer. In our study, we observed variations in TK1 expression among cell lysates, and it is essential to consider various factors that may contribute to lower TK1 levels. TK1 is critical in DNA recycling before cell division and is regarded as a potential marker for prostate cancer prognosis [45]. While the total predictive capacity of TK1 remains unknown, previous research has indicated a correlation between TK1 concentration and prostate cancer-specific mortality [45]. Moving forward, it is essential to acknowledge the limitation of lacking data to confirm TK1 inhibition in less malignant cells. Future research should focus on investigating TK1 regulation and activity in different cellular contexts, particularly exploring its potential inhibitory role in less malignant cells. These forthcoming studies hold promise to offer valuable insights and enhance our comprehension of the intricate role of TK1 in cancer biology. Another limitation of this study is that it did not explore the connection between p53 status and TK1 activity in sEVs. This area presents an opportunity for further investigation to understand better how these factors interact within the context of prostate cancer and sEV biology.

TK1 is located on the outside of the cellular membrane of colorectal, lung, and breast cancer cell lines [16], and cell lines from hematological malignancies, e.g., Raji cells (Burkitt's lymphoma), HL60 cells (promyelocytic leukemia), and Jurkat cells (acute lymphoblastic leukemia) [27]. The upregulation of TK1 in these malignant cells indicates the potential prognostic role of TK1. Interestingly, membrane TK1 seems to be found on the membrane of clinical samples of hematological malignancies like Burkitt's lymphoma, acute promyelocytic leukemia, acute T cell leukemia, and acute lymphoblastic leukemia and shown enzymatic activity, but not detectable in the normal resting or proliferating lymphocytes [46]. Moreover, TK1 is only found on the membrane of malignant cells in colon patient tissue and not on the membrane of healthy normal cells. This may further indicate that TK1's localization to the cell membrane is unique to malignancy [46]. One of the EV subtypes, exosomes, with sizes between 30 and 150 nm in diameter, start their biogenesis with the invagination of the plasma membrane [1], and this could be a way of incorporating the TK1 onto these smaller EVs. This may explain the observed TK1 activity on EVs not undergoing lysis treatment. Tumor cells are reported to exocytose more exosomes into the intercellular space than normal cells [47,48]. Elevated exocytosis, in combination with the upregulated TK1 in tumor cells, may point to the higher enzyme activity we observe in the malignant cell lines used in this study, compared to the prostasomes from healthy males or EV from the regular epithelial cell line.

Here, we demonstrate that EVs derived from highly (PC3) and moderately (DU145) metastatic cells have increased TK1 activity levels compared to that for poor metastatic (LNCaP) and normal epithelial cell line RWPE-1 as well as prostasomes isolated from seminal fluids from healthy male donors. It has been shown that increased TK1 mRNA levels in the plasma-derived sEVs are also associated with clinical resistance to CDK4/6 inhibitors in metastatic breast cancer patients [49], which agrees with our data, and strengthen the plausibility of TK1 as a reliable tool for determining tumor progression.

We have previously shown that patients with prostate cancer have significantly elevated levels of prostasomes in their blood circulation and that the prostasome levels strongly correlate with the Gleason grade of the tumor [50]. It has been shown that isolated EVs contained enriched levels of different proteins, including XIAP, survivin, bFGF, and TK1, essential in cell growth, apoptosis, and angiogenesis. After merging with recipient cells, EVs most likely promote the growth of recipient cells' growth and inhibit apoptosis [51].

Measuring TK1 activity in sEVs holds significant advantages over measuring TK1 activity in plasma. sEVs serve as critical mediators of intercellular communication, carrying biomolecules and signaling molecules. Assessing TK1 activity in sEVs provides valuable insights into its specific involvement in cellular signaling, metastasis, and intercellular communication within these vesicles. This information is pivotal for identifying the disease's origin and understanding TK1's impact in the context of sEV biology. Moreover, sEVs play a crucial role in tumor microenvironment interactions and offer potential as diagnostic and prognostic markers. Measuring TK1 activity in sEVs offers a targeted and specialized approach, yielding unique perspectives on disease mechanisms and potential therapeutic strategies. Our findings on TK1 activity in these EVs could therefore be used to develop an assay that combines the detection of EVs with TK1 activity determination in the EVs. As TK1 enzyme activity is higher in malignant EVs, such an assay would preferably detect prostasomes produced by malignant cells. An assay utilizing an antibody to capture prostasomes and then detection of TK1 activity would be a potentially valuable assay for, e.g., monitoring of prostate cancer, but further studies will be needed. Intriguingly, our investigation also delved into the localization of TK1 within the sEVs enzymes play pivotal roles in sEV function, with many being identified on the vesicle surface. Notably, previous studies have reported the presence of enzymes like Cathepsin B, membrane-type one matrix metalloproteinase (MT1-MMP), and Dipeptidyl peptidase (CD26) on the surface of prostasomes [[52], [53], [54]]. Our study explored TK1's distribution within the sEVs by analyzing its activity before and after sEV lysis (Fig. 4D). The strikingly similar TK1 activity levels before and after lysis hint at a potential surface localization for TK1 within the sEVs. Nevertheless, further topological investigations are warranted in future studies to obtain definitive evidence of TK1's precise localization on the surface of sEV. In this investigation, we employed a real-time PCR-based assay to assess TK1 activity in the samples. The method utilized 5′ exonuclease technology, enabling continuous and real-time monitoring of the enzymatic reaction. Briefly, TK1 catalyzed the conversion of deoxythymidine and ATP to dTMP, releasing dTMP from the probe with a 5′ fluorophore. This event induced fluorescence, allowing for continuous tracking of TK1 activity throughout the reaction. The assay has been previously proved to specifically recognize TK1. This was done by measuring the activity of TK1 and deoxycytidine kinase (dCK) simultaneously, and no significant interference from dCK was observed on the TK1 measurement, and vice versa [55]. Using a homogeneous assay format eliminated the need for washing steps, simplifying the procedure and enhancing assay precision. The real-time PCR approach provided several benefits, including its sensitivity and reliability in accurately measuring TK1 activity [55].

In conclusion, the present study demonstrates that TK1 enzyme activity is highly upregulated in EVs from aggressive metastatic cells but also detectible in EVs from normal cells and seminal prostasomes.

## 4 Methods

### 4.1 Purification of prostasomes

The sample collection and analysis were performed under the guidelines in the Declaration of Helsinki. They were approved by the Ethical Committee of Uppsala University (Ups-01-367), which permitted the use of anonymized surplus materials without informed consent from the donors. Seminal plasma from the Reproductive Centre at Uppsala University Hospital (Uppsala, Sweden) was collected and stored following existing routines [6]. The purified prostasomes, lacking any patient information, were pooled prior to the prostasome purification, and thus not possible to identify the donors in agreement with the ethical permit. All methods were performed following the relevant guidelines and regulations. For the preparation of prostasomes, thawed seminal plasma was centrifuged at 3000×*g* for 12 min at 4 °C. Supernatants were collected and centrifuged at 10,000×*g* for 30 min at 4 °C. Next, supernatants were transferred to new tubes and centrifuged at 100,000×*g* for 2 h at 4 °C using a 90Ti rotor (Beckman Coulter, Brea, CA, USA). The pellets were resuspended in phosphate-buffered saline (PBS), pH 7.6, overnight at 4 °C. Resuspended pellets were next loaded on a chromatography column (XK 60/70, GE Healthcare, Uppsala, Sweden) packed with Superdex 200 gel. Fractions were collected at a flow rate of 5 mL/h. The fractions containing the prostasomes were identified with a spectrophotometer, where peaks at 260 nm (nucleic acid) and 280 nm (proteins, indicating prostasome presence) absorbance were detected. The higher absorbance fractions containing prostasomes were collected, pooled, and centrifuged at 100,000×*g* for 2 h at 4 °C. The obtained pellet was resuspended in PBS and loaded on a sucrose gradient of 1 M, 1.5 M, and 2 M, where 1.5 M is the main EV fraction at 1.13–1.19 g/mL [56]. The gradient was centrifuged at 85,000×*g* for 20–22 h using a SW28.1 rotor (Beckman Coulter). The fractions were collected and centrifuged at 100,000×*g* for 2 h at 4 °C. The pellet was resuspended, and the concentration of prostasomes was adjusted to a total protein concentration of 2 mg/mL, determined using a BCA assay kit (ThermoFischer Scientific, Waltham, Massachusetts, USA). The prostasomes not immediately analyzed were kept at −70 °C until use.

### 4.2 Cell cultures

Three different human prostate cancer cell lines, PC3, DU145, and LNCaP, were cultured, respectively, in DMEM/F-12, RPMI-1640 medium supplemented with 2 mM L-Glu and DMEM GlutaMax (Gibco, ThermoFischer Scientific, USA). All media were supplemented with 100 U/mL penicillin, 100 μg/mL streptomycin, and 10 % fetal bovine serum (FBS; Gibco, Thermo Fischer Scientific, USA). RWPE-1, the prostate epithelial regular cell line, was cultured in Keratinocyte Serum Free Medium (K-SFM), supplemented with bovine pituitary extract (BPE) and human recombinant epidermal growth factor (Gibco, ThermoFischer Scientific, USA). The three tumor cell lines were selected based on the differences in metastatic potential and p53 status. All cell lines were grown and cultured to a 70–80 % confluence. Before EV isolation, cells were washed with PBS, an FBS-free medium was added, and cell culturing was continued for 24 h.

### 4.3 Purification of malignant cell-derived EVs

Cell culture media were harvested from all cell lines and centrifuged at 3000×*g* for 10 min at 4 °C to remove cell debris. The supernatant was collected and filtered through a 0.22 μm filter (Merck Millipore, Burlington, Massachusetts, USA). The filtered media were centrifuged at 112,000×*g* for 2 h at 4 °C using a SW28.1 rotor (Beckman Coulter, USA). The pellets were resuspended in 1 mL PBS containing protease inhibitor (Complete Mini®, Roche, Basel, Schweiz). A second round of centrifugation at 112,000×*g* for 2 h at 4 °C using the same rotor was carried out as the washing step of EV. The resulting EV pellets were resuspended in 200 μL of PBS supplemented with 1x cOmplete™, Mini, EDTA-free Protease Inhibitor Cocktail (Roche), and phosphate inhibitor tablets (PhosSTOP™, Roche). Total protein concentration was determined by the Dot-it-Spot-it assay (Maplestone, Knivsta, Sweden) according to the manufacturer's instructions. Samples were stored at −70 °C until use.

### 4.4 Electron microscopy

Purified, frozen EVs were thawed and mixed with an equal volume of 4 % paraformaldehyde (PFA). A 5 μL sample drop was placed on a formvar and carbon-coated grid. After 20 min, the excess solution was removed by blotting on a filter paper. The sample was washed 3 × 2 min in drops of PBS, transferred to a drop of 1 % glutaraldehyde for 5 min, and washed 8 × 2 min in drops of distilled water. The sample was stained in a drop of uranyl‐oxalate solution (pH 7.0) for 5 min, then stained in a drop of 4 % uranyl acetate (pH 4.0) + 2 % methylcellulose, with a ratio of 1:9 on ice, protected from light for 10 min. The excess uranyl acetate and methylcellulose were removed by blotting on filter paper. Dried grids were examined by transmission electron microscope (TEM), FEI Tecnai™ G2 (ThermoFisher Scientific, USA), operated at 80 kV (Supplementary Fig. 1).

### 4.5 Nanoparticle tracking analysis (NTA)

EVs in ultracentrifuge pellets from PC3, LNCaP, Du145, and RWPE-1 cell lines and Prostasome from seminal fluid samples were analyzed using a Nano sight LM10HSB system equipped for fast video capture and particle-tracking to determine the vesicle size distributions. All 5 EVs samples were diluted in PBS and analyzed in 5 runs each time for 30 s, recorded with a syringe speed of 50 using camera level 10, detection threshold 8, and settings were constant between samples and the auto minimum expected particle size and auto jump distance in analytical software NTA version 3.0 package (Supplementary Fig. 2).

### 4.6 Western blot

Prostate cancer cell lines (PC3, DU145, and LNCaP), their purified EVs, and prostasomes were lysed with lysis buffer (50 mM Tris-HCl (pH 7.4), 150 mM NaCl, 1 mM EDTA, (pH 8), 1 % Triton X-100, 0.1 % sodium deoxycholate and Protease Inhibitor Tablet (Roche complete mini)), pipetted thoroughly, and incubated on ice for 30 min with brief vortexing every 5 min. According to the manufacturer's instructions, total protein concentrations were measured using a Dot-it-Spot-it Total Protein Assay kit (Maplestone AB, Sweden).

To perform western blotting, 10 μg of total protein from each sample was heated to 95 °C for 5 min in 1 x Protein Loading buffer (LI-COR Biosciences) and separated by a 10 % Bis-Tris Gel (Invitrogen) in 1 x NuPAGE™ MES SDS Running Buffer, followed by transfer onto Polyvinylidene fluoride (PVDF) membrane using the iBlot 2 gel transfer device (Invitrogen, ThermoFischer Scientific, USA) according to manufacturer's instructions. Membranes were briefly dried at 37 °C for 5 min, wetted in 1 x TBS for 10 min, and blocked with Intercept® TBS Blocking Buffer (LI-COR) for 60 min. Primary antibodies, including anti-TK1 (Invitrogen, ThermoFischer Scientific, USA), anti-Calnexin (BioLegend, USA), anti-TSG101 (GeneTex, USA), anti-CD9 (Abcam), anti-GAPDH (Cell signaling technologies) and anti-CD63 (Novus Biologicals, USA), were used for Immunoblotting the membranes at 4 °C overnight. The membranes were washed thrice for 5 min in 0.1 % TBS-Tween solution (TBS-T). After that, specific IR Dye 800CW and 680RD fluorophore-conjugated secondary antibody (LI-COR Biosciences) was added to the membranes with dilutions as recommended by the manufacturers and incubated in the dark with gentle shaking at room temperature for 60 min. The membranes were then washed in 0.1 % TBS-T three times for 5 min, and the signals were detected using Odyssey Scanner (LI-COR Biosciences) (Supplementary Fig. 3).

### 4.7 TK1 real-time activity assay

Prostasomes and malignant cell-derived EVs samples were mixed 1:1 with lysis buffer containing complete lysis protease inhibitor (Roche), incubated at room temperature (RT) for 10 min, and vortexed for 2 s. TK1 activity Calibrators to create a TK activity standard curve and samples were mixed with a reagent mix of 1/10. A reagent mix and calibrators were provided by Biovica International AB. The activity is given in TK-REA U/L. One U is equal to 1, 2 × 10^−12^ kat.

Twenty μL reagent or samples mix was transferred to 100 μL PCR tubes (Qiagen, Hilden, Germany) loaded into a Rotor-Gene Q (Qiagen) real-time PCR instrument. Samples and calibrators were run in triplicates. The incubation profile was: 50 min pre-incubation followed by fluorescence data capture every 60 s x 60 at 37 °C. The Rotor-Gene Q software version 2.3.1 (Qiagen) created a run data file. Using this software, raw fluorescence data is next normalized, i.e., set to zero at the start of the fluorescence capture for each sample or calibrator replicate included in the run. Normalized fluorescence units are here denoted Relative fluorescence Units (RfU).

## Funding and additional information

This work was supported by 10.13039/501100004359Swedish Research Council, Sweden (grant number 2020-02258), the Swedish Prostate Cancer Federation, Sweden, 10.13039/100012538Swedish Cancer Foundation, Sweden (grant number 22 2166 Pj), 10.13039/501100009252SciLifeLab, Sweden, AIMDay, Sweden, Lions 10.13039/100002002Cancer Research Foundation, Sweden, and 10.13039/100022734Selander Foundation, United States.

## CRediT authorship contribution statement

**Ehsan Manouchehri Doulabi:** Writing – review & editing, Writing – original draft, Visualization, Methodology, Investigation, Formal analysis, Data curation, Conceptualization. **Louise Dubois:** Writing – review & editing, Writing – original draft, Visualization, Methodology, Formal analysis, Data curation. **Liza Löf:** Writing – review & editing, Methodology, Investigation. **Tanay Kumar Sinha:** Writing – review & editing, Formal analysis. **George Mickhael Harinck:** Writing – review & editing, Data curation. **Per Stålhandske:** Resources, Conceptualization. **Anders Larsson:** Supervision, Project administration, Funding acquisition, Conceptualization. **Masood Kamali-Moghaddam:** Supervision, Resources, Project administration, Conceptualization.

## Declaration of Competing interest

Per Stålhandske is employed by Biovica International AB commercializing the TK1 measurement assay. The other authors state that there is no conflict of interest regarding the publication of this article.

## Appendix A Supplementary data

Multimedia component 1 Multimedia component 1

Multimedia component 2 Supplementary Fig. 1Transmission electron microscopy (TEM) images of cell-derived EVs, **(A)** PC3, **(B)** LNCaP, **(C)** Du145, **(D)** RWPE-1, and **(E)** prostasomes. All five images show the rounded shape of EVs and the bilayered membrane. The size bar at 100 nm indicates that the sEVs of up to 200 nm. Supplementary Fig. 2NTA analysis of the mode particle diameter recorded values of cell-derived EVs from cell lines **(A)** PC3, **(B)** LNCaP, **(C)** Du145, **(D)** RWPE-1, and **(E)** prostasomes. All samples were analyzed in 5 runs. Different EVs showed main peaks in the range of 100–200 nm. Supplementary Fig. 3Representative western blotting results show the presence of EV protein markers, CD63, Calnexin, TK1, CD9 and TSG101, for both cell lines- and EVs lysate. As EV purification control, the endoplasmic reticulum marker, Calnexin, is not present in EVs isolated from cell cultures and seminal fluids but positive in cell line lysate. The presence of TK1 is shown in both sEVs and cell lysates.
