# Supplementary material for: Increased levels of thymidine kinase 1 in malignant cell-derived extracellular vesicles
Source: Biochem Biophys Rep. 2024 Jun 21;39:101761. doi: 10.1016/j.bbrep.2024.101761 (PMC11246012; doi:10.1016/j.bbrep.2024.101761)
Supplement: Multimedia component 1 [file mmc1.docx]

**NANO**SIGHT Ehsan1 2021-03-17 11-06-17

11.80 7.98


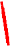

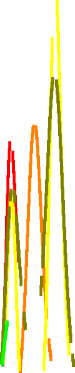

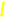

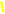

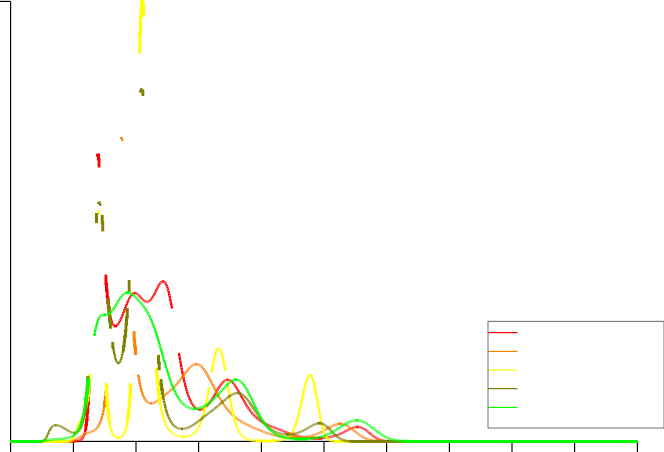


Ehsan1~11-06-31 Ehsan1~11-07-17 Ehsan1~11-08-03 Ehsan1~11-08-51 Ehsan1~11-09-37


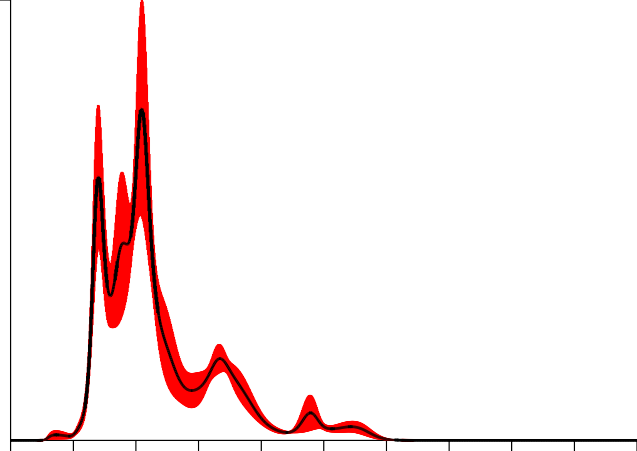


210

141

181

335

479

544

73

Concentration (E6 particles / ml)

Concentration (E6 particles / ml)

0 100 200 300 400 500 600 700 800 900 1000

Size (nm)

FTLA Size / Concentration graph for Experiment: Ehsan1 2021-03-17 11-06-17

0 100 200 300 400 500 600 700 800 900 1000

Size (nm) Averaged FTLA Size / Concentration

Red error bars indicate +/- 1 standard error of the mean

| **Included Files** | **Results** |
| --- | --- |
| Ehsan1 2021-03-17 11-06-31 | Stats: Merged Data |
| Ehsan1 2021-03-17 11-07-17 | Mean: 237.3 nm |
| Ehsan1 2021-03-17 11-08-03 | Mode: 209.2 nm |
| Ehsan1 2021-03-17 11-08-51 | SD: 96.9 nm |
| Ehsan1 2021-03-17 11-09-37 | D10: 139.5 nm |
|  | D50: 209.7 nm |
| **Details** | D90: 367.9 nm |
| NTA Version: NTA 3.0 0060 | Stats: Mean +/- Standard Error |
| Script Used: SOP Standard Measurement 11-06-17AM | Mean: 238.3 +/- 5.0 nm |
| 17Mar2021.txt | Mode: 184.1 +/- 13.0 nm |
| Time Captured: 11:06:17 17/03/2021 | SD: 96.4 +/- 3.4 nm |
| Operator: Ehsan 01 | D10: 143.2 +/- 5.4 nm |
| Pre-treatment: | D50: 210.3 +/- 2.4 nm |
| Sample Name: | D90: 366.2 +/- 4.4 nm |
| Diluent: 2ug/ml | Concentration: 6.70e+008 +/- 4.65e+007 particles/ml |
| Remarks: | 34.0 +/- 2.4 particles/frame |
|  | 40.2 +/- 2.1 centres/frame |
| **Capture Settings** |  |
| Camera Type: SCMOS |  |
| Camera Level: 10 |  |
| Slider Shutter: 600 |  |
| Slider Gain: 250 |  |
| FPS 25.0 |  |
| Number of Frames: 749 |  |
| Temperature: 22.3 oC |  |
| Viscosity: (Water) 0.9 cP |  |
| Dilution factor: Dilution not recorded |  |
| Syringe Pump Speed: 50 |  |
| **Analysis Settings** |  |
| Detect Threshold: 4 |  |
| Blur Size: Auto |  |
| Max Jump Distance: Auto: 7.8 - 9.3 pix |  |

**NANO**SIGHT Ehsan1 2021-03-17 11-30-09

11.61 7.80


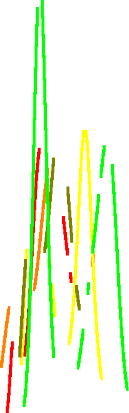

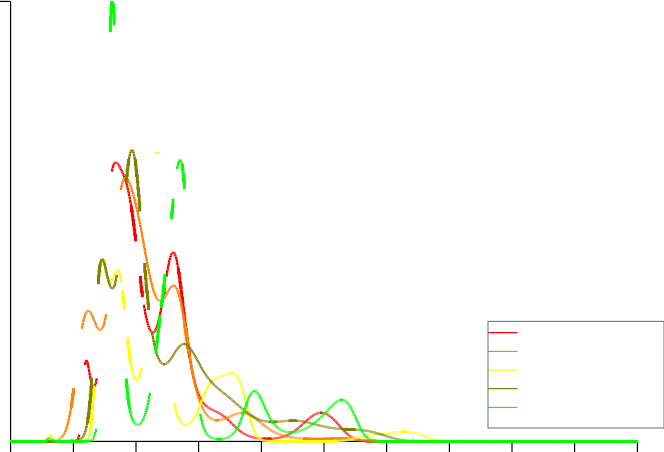


Ehsan1~11-30-39 Ehsan1~11-31-24 Ehsan1~11-32-11 Ehsan1~11-32-56 Ehsan1~11-33-42


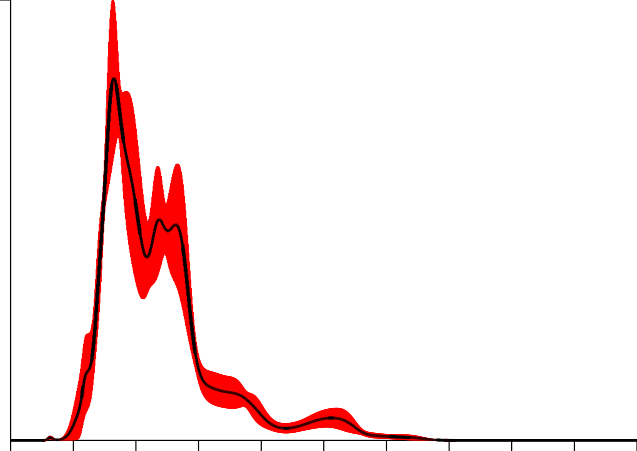


165

238264

513

63

Concentration (E6 particles / ml)

Concentration (E6 particles / ml)

0 100 200 300 400 500 600 700 800 900 1000

Size (nm)

FTLA Size / Concentration graph for Experiment: Ehsan1 2021-03-17 11-30-09

0 100 200 300 400 500 600 700 800 900 1000

Size (nm) Averaged FTLA Size / Concentration

Red error bars indicate +/- 1 standard error of the mean

| **Included Files** | **Results** |
| --- | --- |
| Ehsan1 2021-03-17 11-30-39 | Stats: Merged Data |
| Ehsan1 2021-03-17 11-31-24 | Mean: 237.1 nm |
| Ehsan1 2021-03-17 11-32-11 | Mode: 164.8 nm |
| Ehsan1 2021-03-17 11-32-56 | SD: 94.3 nm |
| Ehsan1 2021-03-17 11-33-42 | D10: 148.3 nm |
|  | D50: 214.8 nm |
| **Details** | D90: 359.1 nm |
| NTA Version: NTA 3.0 0060 | Stats: Mean +/- Standard Error |
| Script Used: SOP Standard Measurement 11-30-09AM | Mean: 237.7 +/- 7.8 nm |
| 17Mar2021.txt | Mode: 188.1 +/- 12.8 nm |
| Time Captured: 11:30:09 17/03/2021 | SD: 92.8 +/- 6.1 nm |
| Operator: prostasome | D10: 146.1 +/- 4.8 nm |
| Pre-treatment: | D50: 218.0 +/- 9.1 nm |
| Sample Name: | D90: 356.5 +/- 23.3 nm |
| Diluent: 2ug/ml | Concentration: 8.25e+008 +/- 4.03e+007 particles/ml |
| Remarks: | 41.9 +/- 2.0 particles/frame |
|  | 48.2 +/- 2.1 centres/frame |
| **Capture Settings** |  |
| Camera Type: SCMOS |  |
| Camera Level: 10 |  |
| Slider Shutter: 600 |  |
| Slider Gain: 250 |  |
| FPS 25.0 |  |
| Number of Frames: 749 |  |
| Temperature: 22.3 oC |  |
| Viscosity: (Water) 0.9 cP |  |
| Dilution factor: Dilution not recorded |  |
| Syringe Pump Speed: 50 |  |
| **Analysis Settings** |  |
| Detect Threshold: 4 |  |
| Blur Size: Auto |  |
| Max Jump Distance: Auto: 7.6 - 9.5 pix |  |

**NANO**SIGHT Ehsan1 2021-03-17 10-49-00

11.38 8.13


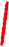

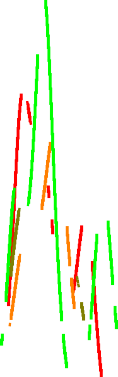

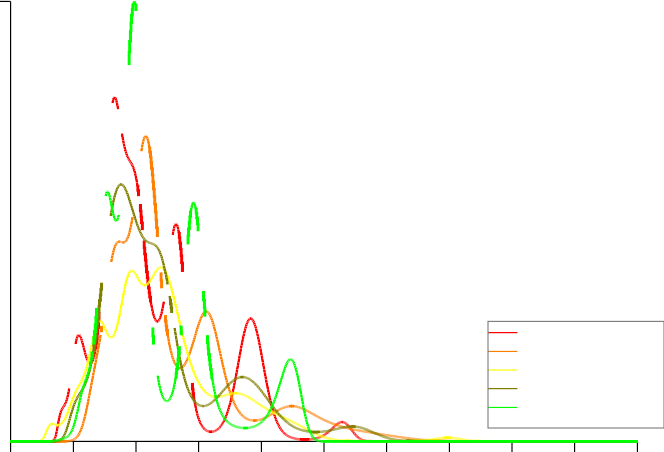


Ehsan1~10-49-35 Ehsan1~10-50-26 Ehsan1~10-51-13 Ehsan1~10-51-59 Ehsan1~10-52-55


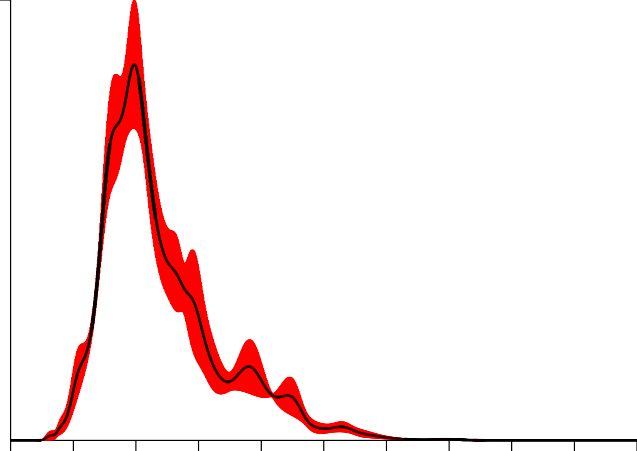


198

381

443

528

696

Concentration (E6 particles / ml)

Concentration (E6 particles / ml)

0 100 200 300 400 500 600 700 800 900 1000

Size (nm)

FTLA Size / Concentration graph for Experiment: Ehsan1 2021-03-17 10-49-00

0 100 200 300 400 500 600 700 800 900 1000

Size (nm) Averaged FTLA Size / Concentration

Red error bars indicate +/- 1 standard error of the mean

| **Included Files** | **Results** |
| --- | --- |
| Ehsan1 2021-03-17 10-49-35 | Stats: Merged Data |
| Ehsan1 2021-03-17 10-50-26 | Mean: 240.0 nm |
| Ehsan1 2021-03-17 10-51-13 | Mode: 197.3 nm |
| Ehsan1 2021-03-17 10-51-59 | SD: 95.5 nm |
| Ehsan1 2021-03-17 10-52-55 | D10: 144.5 nm |
|  | D50: 213.2 nm |
| **Details** | D90: 383.3 nm |
| NTA Version: NTA 3.0 0060 | Stats: Mean +/- Standard Error |
| Script Used: SOP Standard Measurement 10-49-00AM | Mean: 240.3 +/- 4.9 nm |
| 17Mar2021.txt | Mode: 199.0 +/- 13.3 nm |
| Time Captured: 10:49:00 17/03/2021 | SD: 95.0 +/- 2.7 nm |
| Operator: Ehsan | D10: 143.5 +/- 4.6 nm |
| Pre-treatment: | D50: 213.9 +/- 4.9 nm |
| Sample Name: | D90: 384.8 +/- 9.2 nm |
| Diluent: 2ug/ml | Concentration: 1.03e+009 +/- 5.50e+007 particles/ml |
| Remarks: | 52.1 +/- 2.8 particles/frame |
|  | 63.1 +/- 2.5 centres/frame |
| **Capture Settings** |  |
| Camera Type: SCMOS |  |
| Camera Level: 10 |  |
| Slider Shutter: 600 |  |
| Slider Gain: 250 |  |
| FPS 25.0 |  |
| Number of Frames: 749 |  |
| Temperature: 22.3 oC |  |
| Viscosity: (Water) 0.9 cP |  |
| Dilution factor: Dilution not recorded |  |
| Syringe Pump Speed: 50 |  |
| **Analysis Settings** |  |
| Detect Threshold: 4 |  |
| Blur Size: Auto |  |
| Max Jump Distance: Auto: 7.5 - 9.2 pix |  |

**NANO**SIGHT CACO2 DIL50 2019-09-19 11-24-01

12.92 11.31


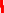

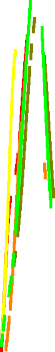

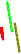

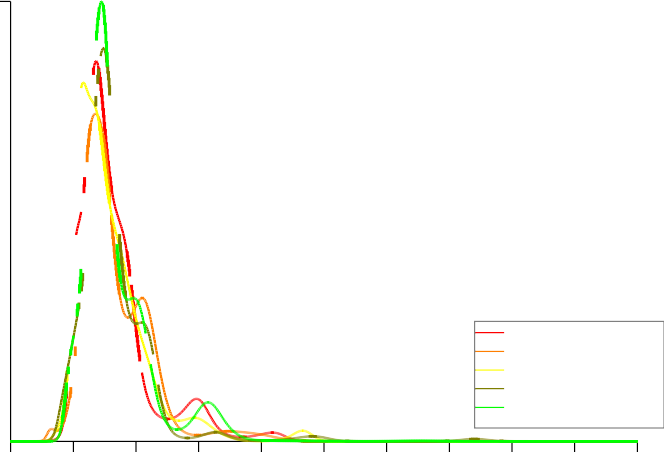


CACO2 D~11-24-19 CACO2 D~11-25-18 CACO2 D~11-26-11 CACO2 D~11-27-07 CACO2 D~11-27-46


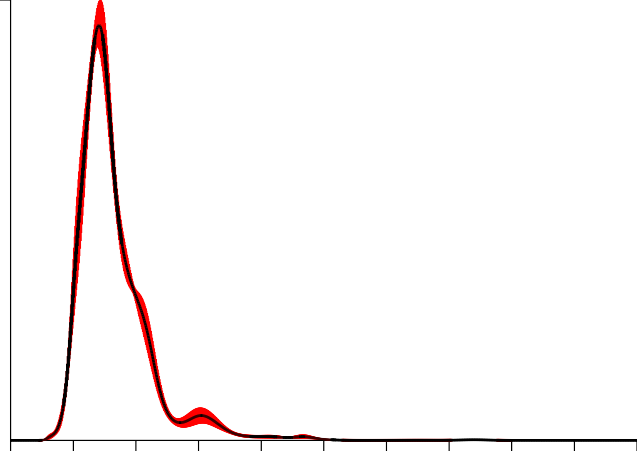


142

305

409 467

656

740

Concentration (E6 particles / ml)

Concentration (E6 particles / ml)

0 100 200 300 400 500 600 700 800 900 1000

Size (nm)

FTLA Size / Concentration graph for Experiment: CACO2 DIL50 2019-09-19 11-24-01

0 100 200 300 400 500 600 700 800 900 1000

Size (nm) Averaged FTLA Size / Concentration

Red error bars indicate +/- 1 standard error of the mean

| **Included Files** | **Results** |
| --- | --- |
| CACO2 DIL50 2019-09-19 11-24-19 | Stats: Merged Data |
| CACO2 DIL50 2019-09-19 11-25-18 | Mean: 164.9 nm |
| CACO2 DIL50 2019-09-19 11-26-11 | Mode: 141.1 nm |
| CACO2 DIL50 2019-09-19 11-27-07 | SD: 60.8 nm |
| CACO2 DIL50 2019-09-19 11-27-46 | D10: 109.4 nm |
|  | D50: 150.0 nm |
| **Details** | D90: 227.7 nm |
| NTA Version: NTA 3.0 0060 | Stats: Mean +/- Standard Error |
| Script Used: SOP Standard Measurement 11-24-01AM | Mean: 165.0 +/- 1.4 nm |
| 19Sep2019.txt | Mode: 136.1 +/- 5.6 nm |
| Time Captured: 11:24:01 19/09/2019 | SD: 60.6 +/- 1.5 nm |
| Operator: Ehsan | D10: 110.1 +/- 1.7 nm |
| Pre-treatment: | D50: 149.8 +/- 1.4 nm |
| Sample Name: CACO2 | D90: 230.8 +/- 4.9 nm |
| Diluent: | Concentration: 9.30e+008 +/- 2.26e+007 particles/ml |
| Remarks: | 47.2 +/- 1.1 particles/frame |
|  | 52.8 +/- 1.0 centres/frame |
| **Capture Settings** |  |
| Camera Type: SCMOS |  |
| Camera Level: 10 |  |
| Slider Shutter: 600 |  |
| Slider Gain: 250 |  |
| FPS 25.0 |  |
| Number of Frames: 749 |  |
| Temperature: 22.0 oC |  |
| Viscosity: (Water) 1.0 cP |  |
| Dilution factor: Dilution not recorded |  |
| Syringe Pump Speed: 50 |  |
| **Analysis Settings** |  |
| Detect Threshold: 3 |  |
| Blur Size: Auto |  |
| Max Jump Distance: Auto: 9.8 - 10.8 pix |  |

**NANO**SIGHT Ehsan1 2021-03-17 11-17-34

9.49 7.74


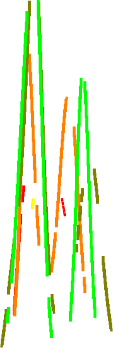

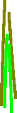

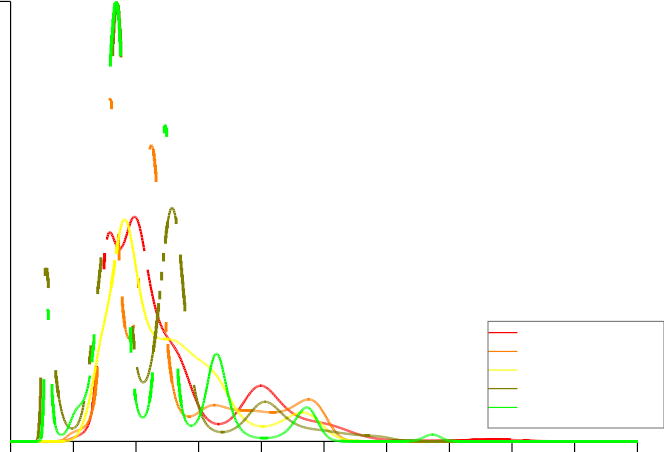


Ehsan1~11-17-56 Ehsan1~11-18-52 Ehsan1~11-19-59 Ehsan1~11-20-57 Ehsan1~11-21-46


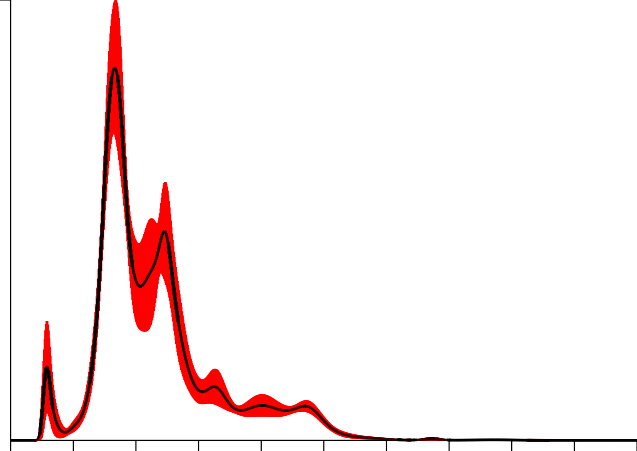


167

246

59

326

402

470

674 772

Concentration (E6 particles / ml)

Concentration (E6 particles / ml)

0 100 200 300 400 500 600 700 800 900 1000

Size (nm)

FTLA Size / Concentration graph for Experiment: Ehsan1 2021-03-17 11-17-34

0 100 200 300 400 500 600 700 800 900 1000

Size (nm) Averaged FTLA Size / Concentration

Red error bars indicate +/- 1 standard error of the mean

| **Included Files** | **Results** |
| --- | --- |
| Ehsan1 2021-03-17 11-17-56 | Stats: Merged Data |
| Ehsan1 2021-03-17 11-18-52 | Mean: 232.0 nm |
| Ehsan1 2021-03-17 11-19-59 | Mode: 166.2 nm |
| Ehsan1 2021-03-17 11-20-57 | SD: 100.8 nm |
| Ehsan1 2021-03-17 11-21-46 | D10: 143.0 nm |
|  | D50: 205.9 nm |
| **Details** | D90: 388.7 nm |
| NTA Version: NTA 3.0 0060 | Stats: Mean +/- Standard Error |
| Script Used: SOP Standard Measurement 11-17-34AM | Mean: 233.7 +/- 8.6 nm |
| 17Mar2021.txt | Mode: 175.3 +/- 6.7 nm |
| Time Captured: 11:17:34 17/03/2021 | SD: 99.0 +/- 3.4 nm |
| Operator: Ehsan 02 | D10: 139.9 +/- 8.5 nm |
| Pre-treatment: | D50: 202.5 +/- 9.0 nm |
| Sample Name: | D90: 381.2 +/- 17.4 nm |
| Diluent: 2ug/ml | Concentration: 7.34e+008 +/- 4.16e+007 particles/ml |
| Remarks: | 37.2 +/- 2.1 particles/frame |
|  | 43.7 +/- 2.9 centres/frame |
| **Capture Settings** |  |
| Camera Type: SCMOS |  |
| Camera Level: 10 |  |
| Slider Shutter: 600 |  |
| Slider Gain: 250 |  |
| FPS 25.0 |  |
| Number of Frames: 749 |  |
| Temperature: 22.3 oC |  |
| Viscosity: (Water) 0.9 cP |  |
| Dilution factor: Dilution not recorded |  |
| Syringe Pump Speed: 50 |  |
| **Analysis Settings** |  |
| Detect Threshold: 4 |  |
| Blur Size: Auto |  |
| Max Jump Distance: Auto: 7.8 - 10.2 pix |  |
