# Supplementary material for: Increased levels of thymidine kinase 1 in malignant cell-derived extracellular vesicles
Source: Biochem Biophys Rep. 2024 Jun 21;39:101761. doi: 10.1016/j.bbrep.2024.101761 (PMC11246012; doi:10.1016/j.bbrep.2024.101761)
Supplement: Multimedia component 2 — Supplementary Fig. 1Transmission electron microscopy (TEM) images of cell-derived EVs, (A) PC3, (B) LNCaP, (C) Du145, (D) RWPE-1, and (E) prostasomes. All five images show the rounded shape of EVs and the bilayered membrane. The size bar at 100 nm indicates that the sEVs of up to 200 nm. Supplementary Fig. 2NTA analysis of the mode particle diameter recorded values of cell-derived EVs from cell lines (A) PC3, (B) LNCaP, (C) Du145, (D) RWPE-1, and (E) prostasomes. All samples were analyzed in 5 runs. Different EVs showed main peaks in the range of 100–200 nm. Supplementary Fig. 3Representative western blotting results show the presence of EV protein markers, CD63, Calnexin, TK1, CD9 and TSG101, for both cell lines- and EVs lysate. As EV purification control, the endoplasmic reticulum marker, Calnexin, is not present in EVs isolated from cell cultures and seminal fluids but positive in cell line lysate. The presence of TK1 is shown in both sEVs and cell lysates. [file mmc2.docx]

**Supplementary Figure 1.** Transmission electron microscopy (TEM) images of cell-derived EVs, **(A)** PC3, **(B)** LNCaP, **(C)** Du145, **(D)** RWPE-1, and **(E)** prostasomes. All five images show the rounded shape of EVs and the bilayered membrane. The size bar at 100 nm indicates that the sEVs of up to 200 nm.

**Supplementary Figure 2.** NTA analysis of the mode particle diameter recorded values of cell-derived EVs from cell lines **(A)** PC3, **(B)** LNCaP, **(C)** Du145, **(D)** RWPE-1, and **(E)** prostasomes. All samples were analyzed in 5 runs. Different EVs showed main peaks in the range of 100 to 200 nm.

**Supplementary Figure 3.** Representative western blotting results show the presence of EV protein markers, CD63, Calnexin, TK1, CD9 and TSG101, for both cell lines- and EVs lysate. As EV purification control, the endoplasmic reticulum marker, Calnexin, is not present in EVs isolated from cell cultures and seminal fluids but positive in cell line lysate. The presence of TK1 is shown in both sEVs and cell lysates.


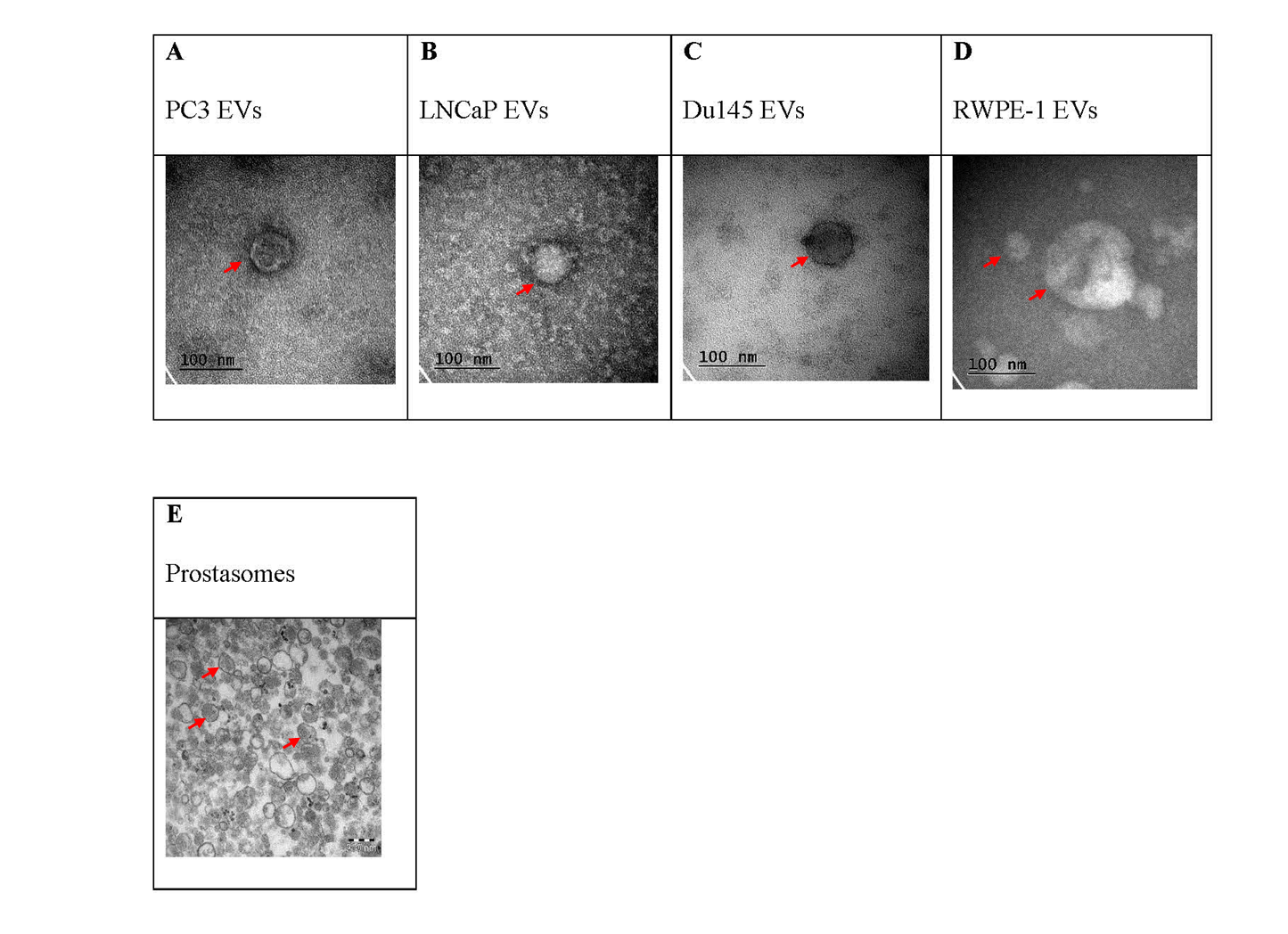


**Supplementary Figure 1.** Transmission electron microscopy (TEM) images of cell-derived EVs, (**A**) PC3, (**B**) LNCaP, (**C**) DU145, (**D**) RWPE-1, and (**E**) SF-EVs. All five images show the rounded shape of EVs and the bilayered membrane. The size bar at 100 nm indicates that the sEVs of up to 200 nm.

**
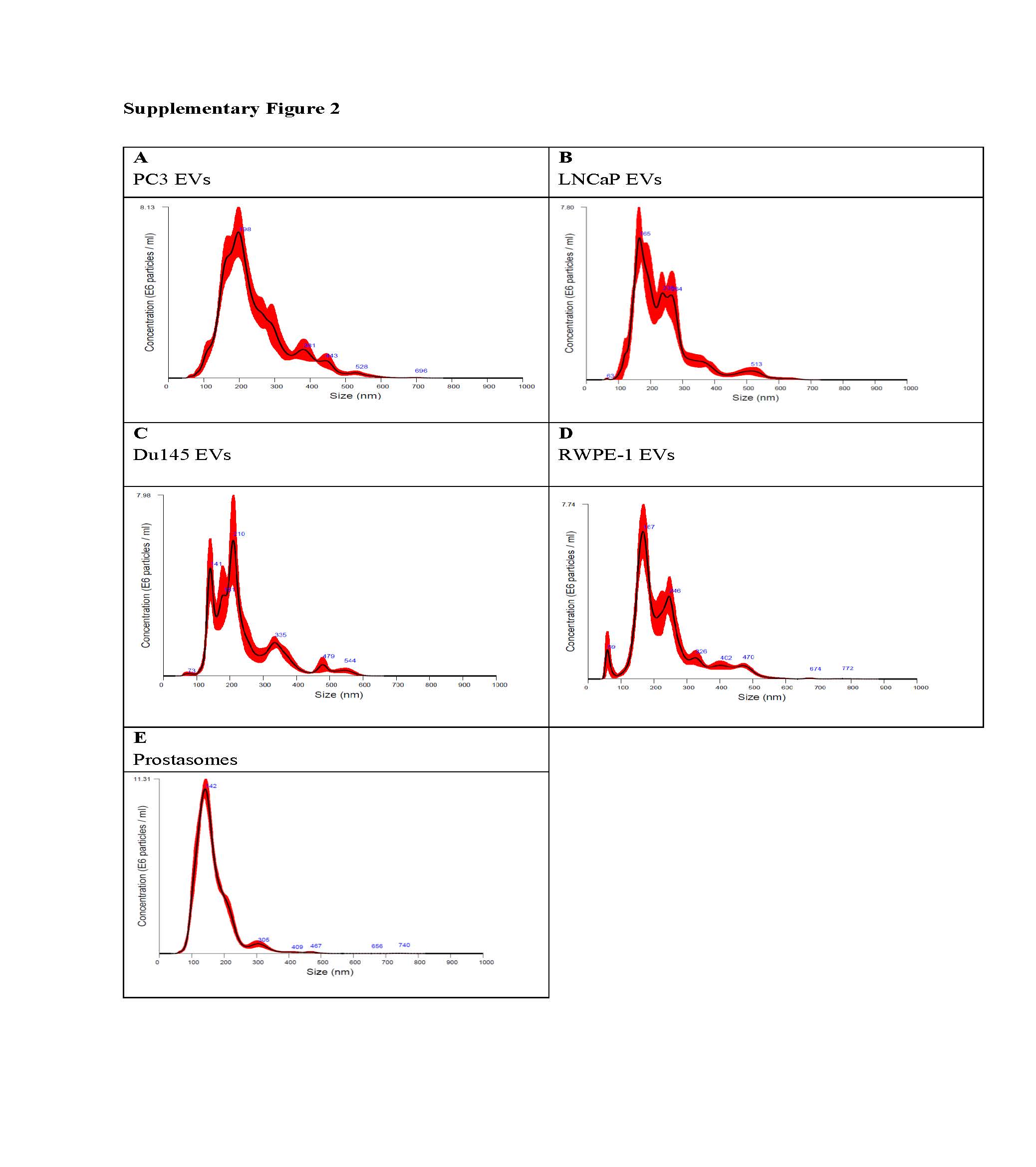
**

**Supplementary Figure 2.** NTA analysis of the mode particle diameter recorded values of cell-derived EVs from cell lines **(A)** PC3, **(B)** LNCaP, **(C)** Du145, **(D)** RWPE-1, and **(E)** prostasomes. All samples were analyzed in 5 runs. Different EVs showed main peaks in the range of 100 to 200 nm.


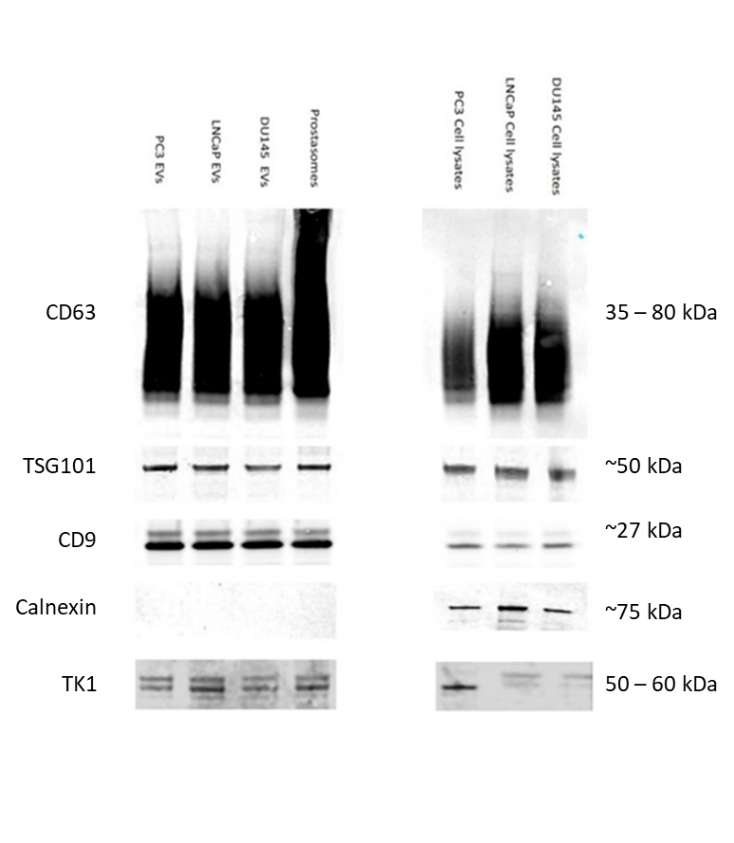


**Supplementary Figure 3.** Representative western blotting results show the presence of EV protein markers, CD63, Calnexin, TK1, CD9 and TSG101, for both cell lines- and EVs lysate. As EV purification control, the endoplasmic reticulum marker, Calnexin, is not present in EVs isolated from cell cultures and seminal fluids but positive in cell line lysate. The presence of TK1 is shown in both sEVs and cell lysates.
